# Evaluation of retinal and choroidal microvasculature parameters by OCTA in patients with premature ovarian insufficiency: a prospective case control study

**DOI:** 10.7717/peerj.21325

**Published:** 2026-06-29

**Authors:** Serdar Ozer, Abdullah Beyoglu, Alev Ozer

**Affiliations:** 1 Department of Obstetrics and Gynecology, Necip Fazil State Hospital, Kahramanmaraş, Turkey; 2 Department of Ophthalmology, Kahramanmaras Sütcü Imam University, Kahramanmaraş, Turkey; 3 Department of Obstetrics and Gynecology, Kahramanmaras Sütcü Imam University, Kahramanmaraş, Turkey

**Keywords:** Microvessels, Premature ovarian insufficiency, Microvessel, Optical coherence tomography angiography, Retina

## Abstract

**Objective:**

Postmenopausal cohorts show reduced vessel density on optical coherence tomography angiography (OCTA), yet OCTA data specific to premature ovarian insufficiency (POI) are lacking. We aimed to characterize retinal and choroidal microvasculature in women with POI using OCTA and to compare these findings with age-matched women with regular menses.

**Methods:**

In this cross-sectional study, 36 women with POI and 52 healthy controls underwent right-eye best-corrected visual acuity testing, Schirmer I, tear breakup time (TBUT), and OCTA (RTVue XR Avanti/AngioVue). Quantitative outputs included macular superficial capillary plexus/deep capillary plexus (SCP/DCP) vessel and flow indices, choriocapillaris flow density, subfoveal choroidal thickness (SFCT), choroidal vascularity index (CVI), and optic nerve head (ONH)/radial peripapillary capillary (RPC) flow area. Imaging was standardized and analyses were masked.

**Results:**

POI participants had significantly lower TBUT (4.41 ± 0.69 *vs* 12.32 ± 2.21 s; *p* = 0.001) and Schirmer scores (6.86 ± 2.20 *vs* 13.76 ± 3.70 mm; *p* < 0.001). SCP/DCP metrics (flow density, non-perfusion, mean and parafoveal vessel density) and retinal nerve fiber layer (RNFL) thickness did not differ between groups (all *p* > 0.05). In contrast, POI showed reduced choriocapillaris flow density (1.75 ± 0.11 *vs* 1.92 ± 0.04 mm^2^; *p* < 0.001), thinner SFCT (282.61 ± 43.50 *vs* 305.36 ± 32.61 µm; *p* = 0.006), and lower macular CVI (0.624 ± 0.04 *vs* 0.699 ± 0.04; *p* = 0.001). At the disc/peripapillary region, ONH vessel density and RPC vessel density were similar (all *p* > 0.05), but flow areas were smaller in POI: ONH flow area (1.59 ± 0.06 *vs* 1.67 ± 0.04 mm^2^; *p* < 0.001), RPC flow area (1.39 ± 0.12 *vs* 1.47 ± 0.18 mm^2^; *p* = 0.011), and choroidal flow area (1.50 ± 0.09 *vs* 1.57 ± 0.09 mm^2^; *p* < 0.001). Temporal peripapillary choroidal thickness was also lower in POI (177.05 ± 28.33 *vs* 211.88 ±  32.89 µm; *p* < 0.001), whereas nasal thickness and nasal/temporal disc CVI were not different (both *p* > 0.05).

**Conclusion:**

To our knowledge, this is the first OCTA study in POI, demonstrating a consistent microvascular pattern—lower choriocapillaris flow, reduced SFCT, and diminished ONH/RPC flow areas—despite preserved SCP/DCP metrics and RNFL thickness. These findings are compatible with estrogen-related microvascular dysregulation and suggest that OCT/OCTA can sensitively detect subclinical ocular perfusion changes in hypoestrogenic states. Confirmation in larger, longitudinal cohorts is needed to establish prognostic value and to test modifiability (*e.g.*, with hormone therapy).

## Introduction

Premature ovarian insufficiency (POI) is defined as hypergonadotropic hypoestrogenism before age 40 and affects approximately 0.1% of women by age 30 and 1% by age 40 (Chon, Umair & Yoon, 2021). Beyond its reproductive implications, POI represents a clinically significant public health concern because it exposes women to prolonged estrogen deficiency during a critical period of cardiometabolic and vascular vulnerability (McGlacken-Byrne & Conway, 2022). The resultant estrogen deficiency is linked to a spectrum of complications—including menstrual irregularity, amenorrhea, infertility, osteopenia, mood disturbances, cognitive changes, sexual dysfunction, diabetes mellitus, and cardiovascular disease (CVD)—with CVD bearing particular relevance for quality of life and life expectancy. Among these sequelae, cardiovascular and microvascular complications are of particular concern, as early endothelial dysfunction may precede overt clinical disease and contribute to long-term morbidity (Ishizuka, 2021; Rezende et al., 2023).

Systemic vascular diseases encompass coronary, cerebrovascular, and peripheral arterial disease, as well as thromboembolic and certain rheumatic or congenital conditions (Joseph et al., 2017). Despite advances in imaging, microvascular pathology remains challenging to assess *in vivo* because of limited direct accessibility (Goldsborough, Osuji & Blaha, 2022). Given that POI is associated with increased cardiovascular risk independent of chronological aging, identifying accessible markers of early microvascular dysfunction is of considerable clinical relevance. The eye offers an accessible window onto the microcirculation: a morphologically intact and well-perfused retina suggests adequate vascular supply, whereas alterations in retinal thickness, texture, or perfusion have been associated with systemic vascular dysfunction (O’Leary & Campbell, 2023; Kellner et al., 2024; Bedggood & Metha, 2020; Matulevičiūė et al., 2022).

Microvascular assessment is particularly relevant in POI because endothelial dysfunction is thought to be an early and potentially reversible consequence of hypoestrogenism. Retinal and choroidal vessels share regulatory mechanisms with systemic vascular beds, including nitric oxide–mediated vasodilation and endothelin-dependent vasoconstriction (Kellner et al., 2024). Therefore, subtle retinal microvascular alterations may reflect broader systemic endothelial imbalance in hypoestrogenic states.

Optical coherence tomography angiography (OCTA) provides noninvasive, depth-resolved visualization of the retinal and choroidal capillary networks, enabling quantitative assessment of perfusion-related indices (*e.g.*, vessel density and flow area) as well as structural vascular parameters (*e.g.*, choroidal vascularity index, CVI) (Javed et al., 2023). For clarity, OCTA-derived metrics can be conceptually grouped into perfusion indices (flow density, flow area, non-perfusion area) and structural vascular indices (subfoveal choroidal thickness (SFCT), choroidal vascularity index (CVI)), each reflecting complementary aspects of microvascular integrity.

Estrogen receptors are present in retinal neurons, glial cells, and vascular endothelium (Wickham et al., 2000). Although the full extent of estrogen’s ocular actions is not completely defined, estrogen deficiency has been implicated in impaired endothelial nitric oxide signaling, heightened vasoconstrictor tone (*e.g.*, endothelin-1), oxidative stress, and reduced endothelial repair capacity (Deschênes et al., 2010). In the retinal and choroidal circulation specifically, estrogen-mediated nitric oxide production contributes to maintenance of choroidal blood flow and autoregulatory stability; thus, estrogen deprivation may disproportionately affect highly perfused vascular beds such as the choriocapillaris and optic nerve head (ONH) microcirculation (Fathy et al., 2022).

In postmenopausal cohorts, several OCTA studies report reductions in macular and optic nerve head (ONH) perfusion, consistent with hypoestrogenic microvascular compromise (Çetinkaya Yaprak & ErkanPota, 2023; Açmaz et al., 2014). However, natural menopause is characterized by a gradual decline in estrogen typically occurring after midlife, often accompanied by age-related vascular changes and comorbidities. Unlike the gradual decline in estrogen during natural menopause, POI entails an abrupt, premature loss of ovarian function before age 40 (Ishizuka, 2021). This distinction makes POI a unique human model to study the vascular consequences of isolated estrogen deficiency relatively independent of chronological aging and accumulated cardiovascular risk.

Evidence in POI to date has largely relied on structural OCT, with only limited work addressing choroidal or retinal thickness changes and, to our knowledge, no systematic characterization of OCTA-derived perfusion metrics in this younger hypoestrogenic population (Sumer et al., 2023). Consequently, whether POI is associated with subclinical microvascular perfusion abnormalities detectable by OCTA remains an unresolved question. This represents a clinically meaningful knowledge gap, as failure to identify early vascular alterations may delay risk stratification and preventive strategies in this population.

Given estrogen sensitivity of the ocular vasculature—particularly within the choriocapillaris and the ONH/radial peripapillary capillary (RPC) plexus—early estrogen deprivation in POI may plausibly disrupt chorioretinal microcirculation. The choriocapillaris supplies the outer retina and photoreceptors and exhibits high metabolic demand, rendering it potentially vulnerable to alterations in endothelial nitric oxide balance. Similarly, the ONH/RPC plexus sustains retinal ganglion cell axons and may be sensitive to perfusion instability in hypoestrogenic states.

We aimed to quantify choriocapillaris flow, subfoveal choroidal thickness (SFCT), ONH/RPC perfusion (flow area), and CVI in POI *versus* age-matched controls using standardized OCTA with masked analysis. We hypothesized that women with POI would demonstrate reduced perfusion-related indices (choriocapillaris flow and ONH/RPC flow area) and structural vascular alterations (SFCT and CVI), reflecting early hypoestrogenism-associated microvascular dysregulation.

## Materials and Methods

### Study design and setting

This prospective case–control study was conducted at the Departments of Ophthalmology and Gynecology, Kahramanmaraş Sütçü İmam University Hospital, between January 2021 and January 2023. Ethical approval was obtained from the local ethics committee (Approval No. 2020/10), and written informed consent was obtained from all participants in accordance with the Declaration of Helsinki.

A prospective case–control design was chosen to enable standardized, contemporaneous assessment of microvascular parameters in women with POI and age-matched healthy controls under identical imaging conditions. Given the relatively low prevalence of POI, this design allowed feasible comparison of clearly defined hypoestrogenic and normoestrogenic groups while minimizing temporal and measurement variability.

Thirty-six women diagnosed with POI and 52 age-matched healthy controls with regular menstrual cycles were included. POI was defined as amenorrhea or oligomenorrhea for >4 months with follicle-stimulating hormone (FSH) levels >25 mIU/mL on two separate occasions at least 4 weeks apart.

All POI diagnoses were confirmed by experienced gynecologists based on established diagnostic criteria, and secondary causes of ovarian insufficiency—including pregnancy, thyroid dysfunction, hyperprolactinemia, genetic conditions, autoimmune disorders, or prior ovarian surgery/chemotherapy—were systematically excluded during clinical evaluation.

Hormonal measurements (FSH and estradiol) were retrieved from electronic medical records at the time of POI diagnosis. Hormonal assays were not routinely performed in the control group because all controls had regular menstrual cycles and no clinical suspicion of estrogen deficiency. Although biochemical confirmation was not available in controls, the likelihood of occult POI in women with regular cycles is considered low; nevertheless, potential minimal misclassification cannot be completely excluded.

Inclusion criteria required fulfillment of POI diagnostic criteria for the case group and regular menstrual cycles without systemic or ocular disease for controls. Exclusion criteria were grouped to minimize confounding:

• Systemic conditions: Smoking (active or passive), hypertension, diabetes mellitus, cardiovascular or renal disease, metabolic syndrome, obstructive sleep apnea.
• Medication-related factors: Use of hormonal therapy, oral contraceptives, hormone replacement therapy, or medications affecting vascular parameters.
• Ocular conditions: Refractive error >±2 diopters, cataract, glaucoma, retinal disease, optic neuropathy, uveitis, prior ocular surgery or trauma.

**Figure 1:**
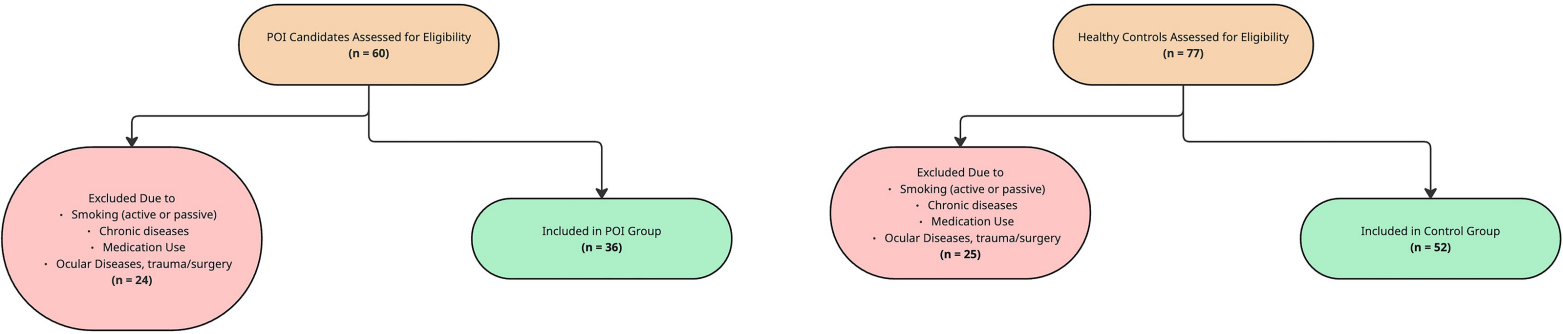
Participant flow diagram for study enrollment and group allocation.

Sixty women with POI and 77 potential controls were screened. Among POI candidates, 24 were excluded (17 due to predefined systemic or ocular comorbidities and seven declined participation), yielding 36 participants. Among controls, 25 were excluded for similar reasons, yielding 52 participants (Fig. 1).

Tear assessment was performed after OCTA imaging to avoid test-induced artifacts. Schirmer I testing was conducted with filter paper strips placed in the lower fornix for 5 min with eyes closed; wetting length was recorded in millimeters. Tear breakup time (TBUT) was measured after fluorescein instillation under cobalt-blue illumination.

Tear evaluation was included because estrogen deficiency is known to affect ocular surface physiology, and severe tear instability could theoretically influence OCTA signal quality.

### OCTA acquisition protocol

All scans were acquired using RTVue XR Avanti (Optovue Inc., Fremont, CA, USA; AngioVue software v2018.0.0.18) by a single experienced examiner (A.B.) under standardized room conditions between 09:30 and 10:00 AM to minimize diurnal variation. Only the right eye of each participant was analyzed to avoid statistical inter-eye correlation and potential bias from arbitrary eye selection.

**Figure 2:**
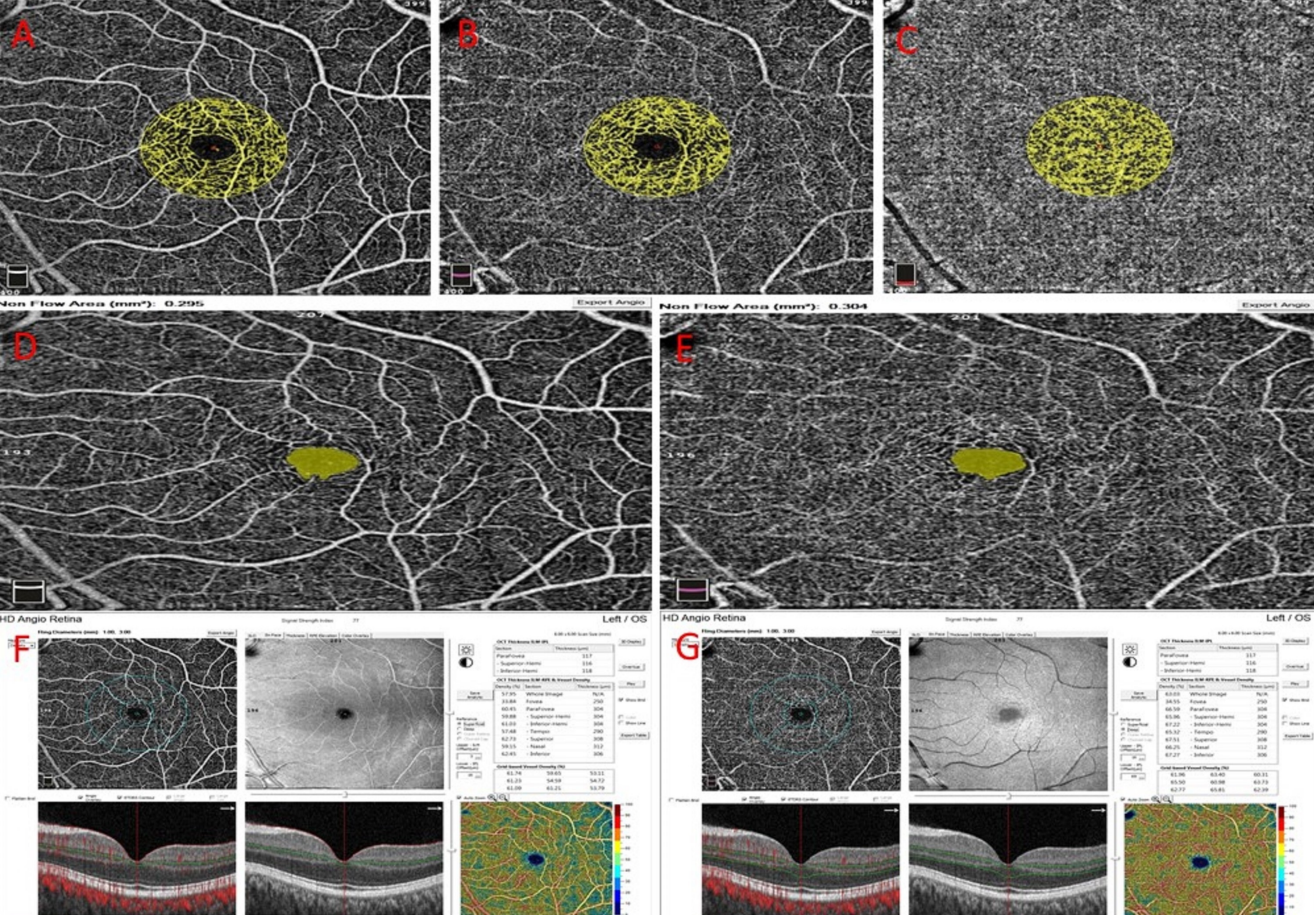
Optic chorence tomography angiography macular measurument. This software automatically calculated blood flow in the superficial (A), deep (B), and choriocapillaris (C) layers of the macula. The low blood flow region located in the center of the macula was defined as the foveal avascular area (FAZ). The software automatically calculated the superficial (D) and deep (E) layers. Vascular density (VD) was defined as the percentage of vessels and microvessels in a selected region. The mean VD value in the superficial (F) and deep (G) layers was automatically measured.

**Figure 3:**
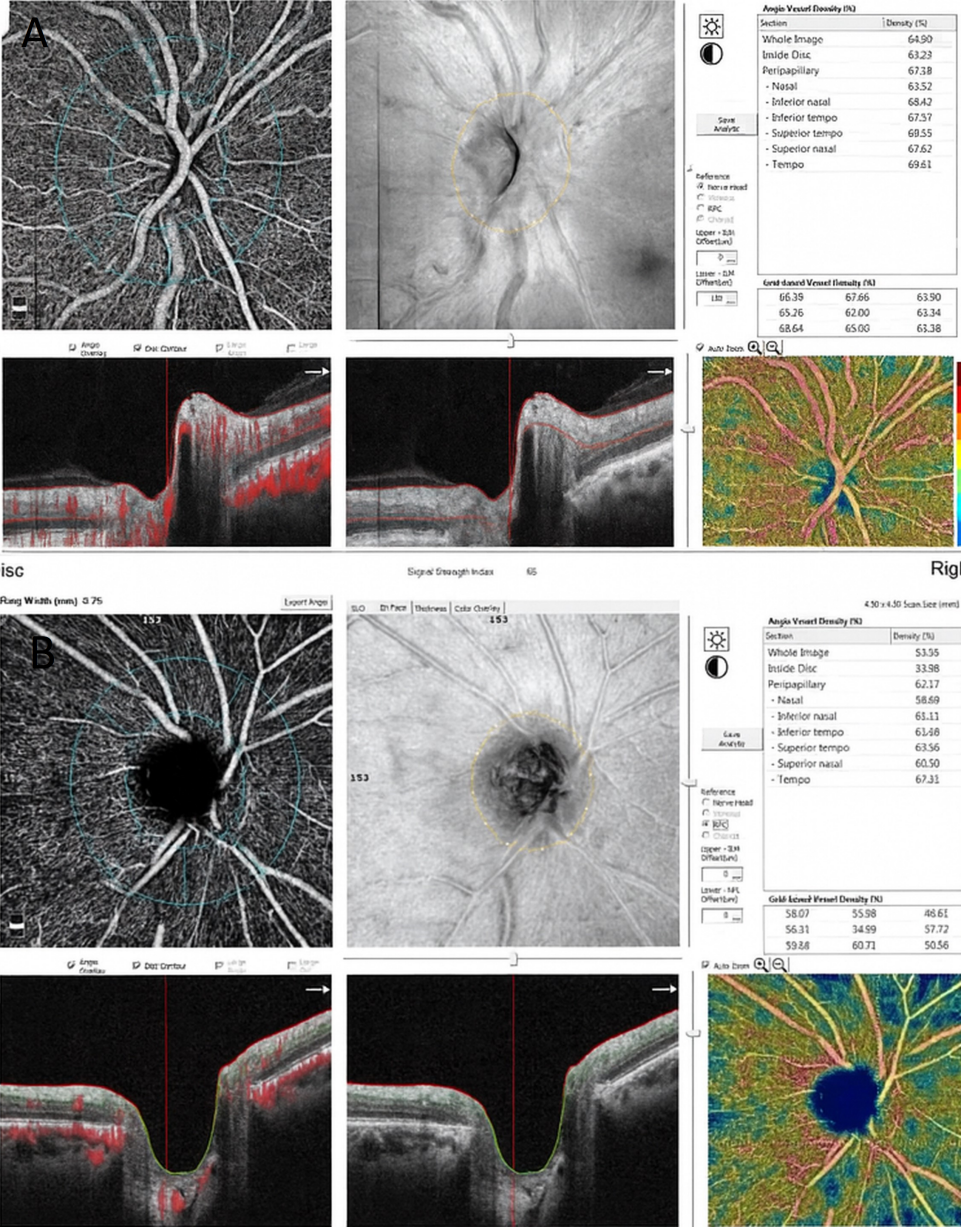
Optic nerve head (ONH) and radial peripapillary capillary (RPC) density measurement multimodal analysis of OCTA peripapillary vessel density in ONH mode (A) and RPC mode (B) was automatically measured by the device software. Multimodal analysis of OCTA peripapillary vessel density in ONH mode (A) and RPC mode (B) was automatically measured by the device software.

Two volumetric scans were obtained per eye:

• A 6 × 6 mm macular scan (Fig. 2)
• A 4.5 × 4.5 mm optic nerve head (ONH) scan (Fig. 3)

The ONH volume comprised 304 B-scans equally spaced on both axes, each containing 304 A-scans. The macular volume comprised 400 B-scans, each containing 400 A-scans.

Each scan was repeated after a 15-minute rest period; the average of the two measurements was used for analysis. In a pilot subset (*n* = 20), intra-session reproducibility analysis demonstrated high agreement, with intraclass correlation coefficients (ICC) >0.85 for primary perfusion parameters.

Images with segmentation failure, motion artifacts, projection artifacts, or signal strength index <50 (manufacturer-recommended threshold) were excluded.

Device-defined segmentation slabs included:

• Superficial capillary plexus (SCP): from internal limiting membrane (ILM) to 10 µm above inner plexiform layer (IPL)
• Deep capillary plexus (DCP): from 10 µm above IPL to 10 µm below outer plexiform layer (OPL)
• Radial peripapillary capillary (RPC): from ILM to posterior boundary of the retinal nerve fiber layer (RNFL)
• Choriocapillaris slab as defined by manufacturer (Archer, Gardiner & Stitt, 2007).

Segmentation boundaries followed manufacturer defaults to ensure reproducibility and comparability with prior OCTA literature, acknowledging that customized segmentation may yield slightly different absolute values.

ONH and RPC flow area metrics were exported directly from the device under default motion correction and projection-artifact removal algorithms; no external thresholding was applied to these vendor-derived perfusion outputs.

### Quantitative metrics and regional analysis

Vessel density (%) was defined as the proportion of the scanned area occupied by perfused vasculature within a given region. Flow area (mm^2^) represented the absolute area occupied by detectable flow signal within a specified slab.

Regional analyses were performed using the device’s ETDRS-like grid centered on the fovea (macular scan) and on the optic disc (ONH scan), divided into superior-nasal, inferior-nasal, superior-temporal, and inferior-temporal quadrants (Matsunaga et al., 2014). Quadrant-based analysis was selected to detect potential regional susceptibility patterns across vascular territories.

#### Subfoveal choroidal thickness

SFCT was measured manually on enhanced-depth imaging scans as the perpendicular distance from the outer border of the retinal pigment epithelium (RPE) to the choroid–scleral junction at the foveal center by two masked graders. The mean of the two measurements was analyzed. Interobserver agreement demonstrated strong concordance (ICC = 0.89). Discrepancies >10 µm were resolved by joint review.

#### Choroidal vascularity index

**Figure 4:**
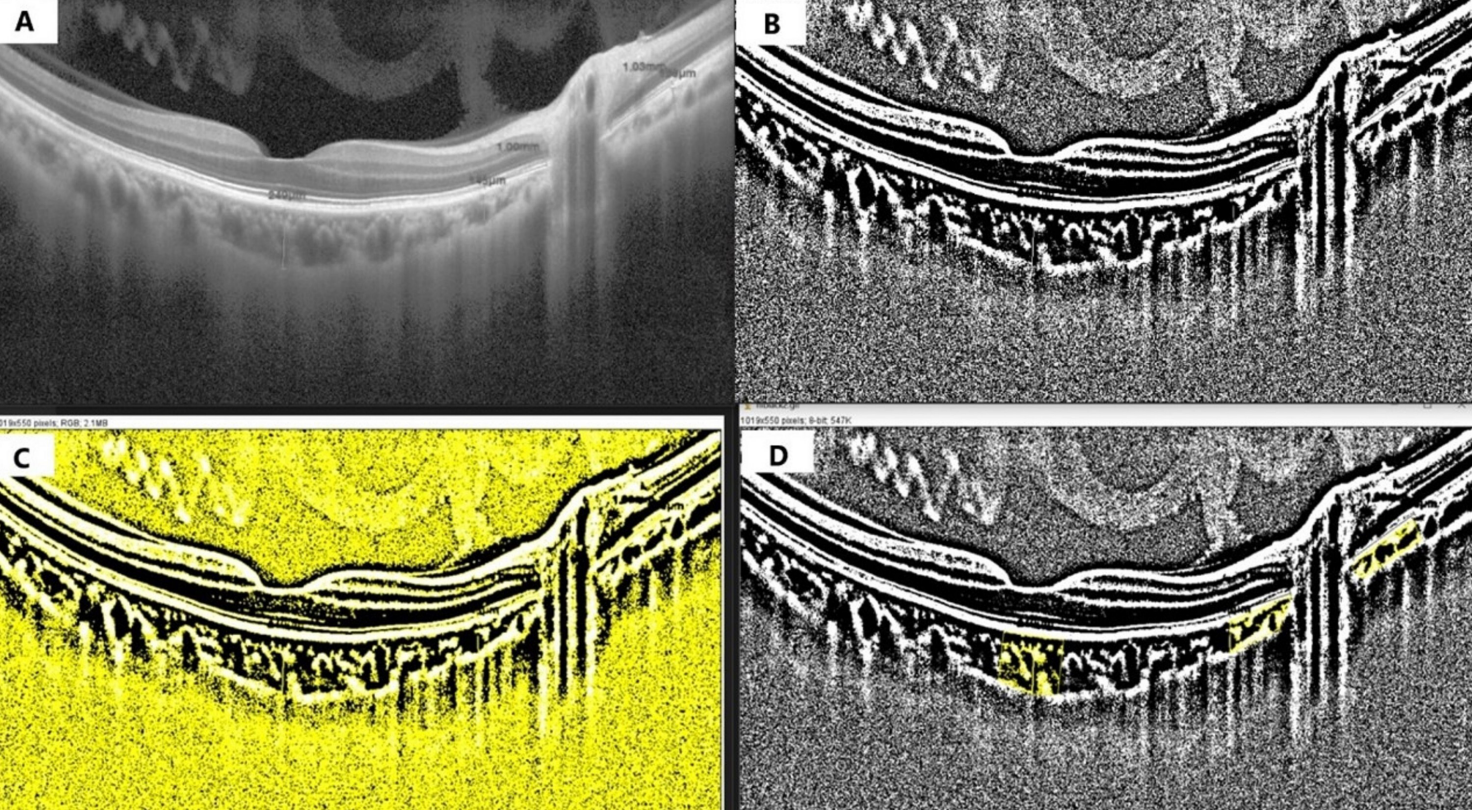
Calculation of choroidal vascularity index. The image was converted to 8-bit format using ImageJ (A) and binaryized using Niblack’s automatic local threshold (B). It was then converted to red, green, and blue (RBG) color, and the area of dark pixels was defined as the luminal area (C). Luminal and total choroidal areas were calculated using ROI segmentation with the software (D).

CVI was calculated as the ratio of luminal area (LA) to total choroidal area (TCA) on binarized enhanced-depth imaging scans using ImageJ v1.53 (Fig. 4).

Processing steps included:

1. Manual delineation of TCA between the RPE and choroid–scleral junction.
2. Conversion to 8-bit format and binarization using Niblack local thresholding (radius = 25 pixels; k = −0.2).
3. Isolation of luminal area *via* color thresholding.
4. Computation of CVI = LA/TCA (Agrawal et al., 2016).

Alternative thresholding methods (Otsu global thresholding and Phansalkar local thresholding) were evaluated in sensitivity analyses; however, Otsu tended to over-segment luminal areas in shadowed regions, and Phansalkar demonstrated greater inter-scan variability. Therefore, Niblack thresholding was retained for primary analysis.

For quality assurance, a subset of images was cross-checked against manual binarization to confirm agreement with vendor-derived perfusion metrics.

#### Quality assurance

All imaging protocols, segmentation settings, and analysis pipelines were identical for cases and controls. Image analysis was performed by a masked examiner using standardized procedures. Periodic cross-checks of approximately 10% of the dataset were conducted to ensure consistency and resolve discrepancies.

#### Statistical analysis

Statistical analyses were performed using SPSS v22.0 (IBM Corp., Armonk, NY, USA). Data distribution was assessed using Kolmogorov–Smirnov testing and Q–Q plots. Continuous variables are presented as mean ± standard deviation. Group comparisons were conducted using two-sided Student’s *t*-tests or chi-square tests where appropriate, with significance set at *p* < 0.05.

Multivariable adjustment was not performed because strict exclusion criteria and age/BMI matching were applied to minimize confounding; however, residual confounding cannot be completely excluded.

A formal *a priori* power calculation was not feasible due to the limited number of eligible POI cases; therefore, the moderate sample size may increase the risk of type II error, particularly for small effect sizes.

## Results

Table 1 summarizes demographic and clinical characteristics. The two groups were comparable with respect to age, gravidity, parity, body mass index, and best-corrected visual acuity, indicating successful matching and minimizing major demographic confounding (detailed values are presented in Table 1).

**Table 1:** Demographic and clinical characteristics of the participants.

	Primary ovarian insufficiency (*n* = 36)	Healthy controls (*n* = 52)	*P* value
Age (years)	35.91 ± 3.54	35.63 ± 3.44	0.712
Gravida (n)	1.47 ± 0.65	1.44 ± 0.77	0.846
Parity (n)	1.08 ± 0.69	1.28 ± 0.74	0.190
Body mass index (kg/m^2^)	26.69 ± 4.17	26.44 ± 4.56	0.797
FSH (IU/L)	60.4 ± 19.4	–	–
Estradiol (IU/L)	19.5 ± 8.5	–	–
Visual acuity of right eye	0.99 ± 0.23	0.99 ± 0.23	0.966
Tear film breakup time (sec)	4.41 ± 0.69	12.32 ± 2.21	0.001^*^
Schirmer test score (mm)	6.86 ± 2.20	13.76 ± 3.70	0.001^*^

**Notes.**

* *p* < 0.05 was accepted to be statistically significant.

All values were expressed as mean ± standart deviation.

Mean FSH and estradiol levels in the POI group confirmed a hypergonadotropic hypoestrogenic state (FSH 60.4 ± 19.3 mIU/mL; estradiol 19.5 ± 8.5 pg/mL). Hormonal assays were not performed in the control group, consistent with the predefined study protocol, as all controls had regular menstrual cycles and no clinical evidence of estrogen deficiency.

POI participants demonstrated significantly lower TBUT and Schirmer test scores compared with controls (both *p* = 0.001). These tear parameters were assessed because estrogen deficiency may influence ocular surface physiology and potentially affect OCTA image quality; their reduction in POI supports the presence of a hypoestrogenic ocular phenotype.

### Retinal (macular) OCTA findings

As shown in Table 2, macular retinal plexus metrics (superficial and deep capillary plexus) did not differ significantly between POI and controls across vessel density (%) and flow-related parameters (mm^2^), indicating preserved retinal microvascular density in both groups.

**Table 2:** Retinal optical coherence tomography angiography findings in participants.

	Primary ovarian insufficiency (*n* = 36)	Healthy controls (*n* = 52)	*P* value
Superficial capillary plexus			
Flow density (mm^2^)	1.40 ± 0.08	1.40 ± 0.09	0.887
Non-perfusion (mm^2^)	0.37 ± 0.09	0.33 ± 0.10	0.125
Vessel density (mean) (%)	49.61 ± 2.03	50.29 ± 2.47	0.181
Vessel density (parafoveal) (%)	52.57 ± 2.20	53.83 ± 3.63	0.068
Deep capillary plexus			
Flow density (mm^2^)	1.45 ± 0.12	1.50 ± 0.13	0.095
Non-perfusion (mm^2^)	0.41 ± 0.10	0.38 ± 0.13	0.242
Vessel density (mean) (%)	57.60 ± 3.21	57.25 ± 4.22	0.672
Vessel density (parafoveal) (%)	62.41 ± 2.56	62.35 ± 3.21	0.926
Choriocapillaris			
Flow density (mm^2^)	1.75 ± 0.11	1.92 ± 0.04	<0.001^*^
Choroidal thickness			
Subfoveal Choroidal (µn)	282.61 ± 43.50	305.36 ± 32.61	0.006^*^
Vascular index			
Choroidal Vascular Index	0.624 ± 0.04	0.699 ± 0.04	<0.001^*^

**Notes.**

* *p* < 0.05 was accepted as statistically significant.

All values were expressed as mean ± standart deviation.

Specifically, no statistically significant differences were observed in SCP or DCP flow density (expressed in mm^2^) or vessel density (expressed as percentage area occupied by perfused vasculature) (all *p* > 0.05; see Table 2 for detailed values).

In contrast, choroidal parameters demonstrated consistent and statistically robust differences. Choriocapillaris flow density was significantly lower in POI (1.75 ± 0.11 *vs* 1.92 ± 0.04 mm^2^; *p* < 0.001), representing approximately a 9% relative reduction compared with controls. Subfoveal choroidal thickness (SFCT) was also reduced in POI (282.61 ± 43.50 *vs* 305.36 ± 32.61 µm; *p* = 0.006), corresponding to an approximate 7% decrease in mean thickness.

Similarly, macular choroidal vascularity index (CVI) was lower in POI (0.624 ± 0.04 *vs* 0.699 ± 0.04; *p* < 0.001), reflecting a reduced proportion of luminal (vascular) area within total choroidal tissue.

Taken together, these findings demonstrate a clear dissociation: while retinal capillary plexus density remains preserved, choroidal perfusion and structural vascular indices are significantly reduced in women with POI.

### Optic disc and peripapillary OCTA findings

Table 3 summarizes optic disc and peripapillary results. Overall, vessel density (%) parameters at the optic nerve head (ONH) and radial peripapillary capillary (RPC) plexus did not differ significantly between groups. However, flow area metrics (expressed in mm^2^), which reflect absolute perfused area rather than proportional density, were consistently reduced in POI.

**Table 3:** Optic disc optical coherence tomography angiography parameters in group.

	Primary ovarian insufficiency (*n* = 36)	Healthy controls (*n* = 52)	*P* value
Vessel density (%)			
Optic nerve head (mean)	62.64 ± 2.57	62.01 ± 2.88	0.296
Optic nerve head (superior nasal)	62.46 ± 4.74	62.34 ± 3.17	0.886
Optic nerve head (inferior nasal)	60.77 ± 3.61	60.45 ± 3.54	0.682
Optic nerve head (nasal)	64.62 ± 4.41	64.59 ± 2.94	0.961
Optic nerve head (superior temporal)	64.17 ± 3.95	64.67 ± 3.18	0.515
Optic nerve head (inferior temporal)	66.61 ± 3.58	66.03 ± 2.89	0.404
Optic nerve head (temporal)	61.45 ± 3.33	61.74 ± 4.08	0.729
Disc flow (mm^2^)			
Optic Nerve Head Flow Area	1.59 ± 0.06	1.67 ± 0.04	<0.001^*^
Radial Peripapillary Flow Area	1.39 ± 0.12	1.47 ± 0.18	<0.011^*^
Choroidal Flow Area	1.50 ± 0.09	1.57 ± 0.09	<0.001^*^
Radial peripapillary capillary plexus (%)			
Vessel density at optic nerve head (mean)	63.82 ± 2.47	64.23 ± 3.00	0.502
Vessel density at optic nerve head (superior nasal)	62.31 ± 4.76	62.79 ± 4.09	0.615
Vessel density at optic nerve head (inferior nasal)	65.99 ± 4.68	65.96 ± 2.82	0.970
Vessel density at optic nerve head (nasal)	60.60 ± 3.56	60.88 ± 4.79	0.762
Vessel density at optic nerve head (superior temporal)	67.17 ± 3.91	67.36 ± 3.62	0.812
Vessel density at optic nerve head (inferior temporal)	68.90 ± 3.83	69.66 ± 2.01	0.234
Vessel density at optic nerve head (temporal)	64.33 ± 3.17	64.61 ± 3.29	0.686
Optic disc choroidal thickness (µn)			
Nasal choroidal	171.72 ± 32.85	186.32 ± 38.79	0.068
Temporal choroidal	177.05 ± 28.33	211.88 ± 32.89	<0.001^*^
Optic disc vascular index			
Nasal choroidal	0.651 ± 0.05	0.662 ± 0.03	0.252
Temporal chroidal	0.661 ± 0.06	0.669 ± 0.02	0.423
RNFT (µn)	91.64 ± 15.38	91.56 ± 6.34	0.973

**Notes.**

* *p* < 0.05 was accepted as statistically significant.

All values were expressed as mean ± standart deviation. RNFT, Retinal nerve fiber thickness.

ONH flow area was lower in POI (1.59 ± 0.06 *vs* 1.67 ± 0.04 mm^2^; *p* < 0.001), corresponding to an approximate 5% reduction. Radial peripapillary flow area was also reduced (1.39 ± 0.12 *vs* 1.47 ± 0.18 mm^2^; *p* = 0.011), as was choroidal flow area at the disc (1.50 ± 0.09 *vs* 1.57 ± 0.09 mm^2^; *p* < 0.001).

This pattern mirrors the macular findings: proportional vessel density remains stable, whereas absolute perfusion-related flow areas are diminished in POI.

Optic-disc choroidal thickness showed a regional asymmetry. Temporal peripapillary choroidal thickness was significantly lower in POI (177.05 ± 28.33 *vs* 211.88 ± 32.89 µm; *p* < 0.001), whereas the nasal sector did not differ significantly (*p* = 0.068). This temporal–nasal asymmetry may indicate region-specific susceptibility of the temporal peripapillary choroid, which anatomically corresponds to the papillomacular bundle region.

Disc CVI (nasal and temporal sectors) did not differ significantly between groups (both *p* > 0.05).

Importantly, RNFL thickness did not differ between POI and controls (91.64 ± 15.38 *vs* 91.56 ± 6.34 µm; *p* = 0.973). The preservation of RNFL thickness, despite reduced perfusion-related parameters, reinforces the dissociation between early microvascular alterations and overt structural neural damage.

In summary, women with POI exhibited a coherent microvascular profile characterized by preserved retinal vessel density but reduced choroidal perfusion, decreased choroidal thickness, and diminished ONH/RPC flow areas. Structural neural thickness parameters remained stable, suggesting that perfusion abnormalities may precede measurable neurostructural changes in this hypoestrogenic population.

## Discussion

In this prospective case–control study, women with POI demonstrated a consistent OCTA pattern characterized by reduced choriocapillaris flow, decreased subfoveal choroidal thickness, and diminished ONH/RPC flow areas compared with age-matched controls. In contrast, superficial and deep retinal capillary plexus vessel densities and RNFL thickness were preserved.

This compartment-specific dissociation between impaired choroidal/peripapillary perfusion and preserved retinal capillary density and neural thickness represents the central finding of the study.

POI participants also exhibited lower TBUT and Schirmer scores, consistent with an estrogen-deficient ocular surface phenotype. Although tear film instability may theoretically influence OCTA signal quality, imaging was performed prior to tear testing, low-quality scans were excluded based on predefined signal strength criteria, and the directionality and internal consistency of perfusion findings across independent vascular beds make a purely artifact-driven explanation unlikely. Nevertheless, residual confounding cannot be completely excluded.

Our perfusion-focused findings extend prior structural observations in POI. Sumer et al. (2023) reported reduced choroidal thickness and ocular surface alterations in women with premature ovarian failure. Our results build upon these observations by demonstrating that perfusion-related OCTA metrics—particularly choriocapillaris flow and ONH/RPC flow areas—are altered even when retinal capillary density and RNFL thickness remain unchanged.

In postmenopausal cohorts, OCTA studies have documented reduced macular and ONH perfusion (Fathy et al., 2022; Çetinkaya Yaprak & ErkanPota, 2023). However, natural menopause typically occurs in later life and is frequently accompanied by age-related vascular remodeling and cardiometabolic comorbidities. In contrast, POI represents premature estrogen deprivation in relatively young women, allowing partial separation of hypoestrogenism from chronological aging. Our findings suggest that estrogen-related microvascular alterations may manifest independently of advanced age.

The preservation of SCP/DCP vessel density alongside reduced choriocapillaris and ONH/RPC flow areas suggests early, compartment-specific microvascular vulnerability in POI. The choriocapillaris is characterized by high metabolic demand and limited autoregulatory reserve, supplying the outer retina and photoreceptors. The ONH/RPC plexus sustains retinal ganglion cell axons and is similarly dependent on stable perfusion. These vascular territories may therefore be more sensitive to subtle endothelial imbalance induced by estrogen deficiency than the inner retinal plexus.

Estrogen exerts vasodilatory and endothelial-protective effects through nitric oxide modulation, attenuation of endothelin-1 signaling, and antioxidant activity (Aryan et al., 2020; Guivarc’h et al., 2015; Zhu et al., 2002). Estrogen receptors are expressed in retinal neurons, glial cells, and vascular endothelium (Deschênes et al., 2010; Vajaranant & Pasquale, 2012). Within the choroidal circulation, estrogen-mediated nitric oxide production contributes to maintenance of choroidal blood flow; reduced estrogen may shift this balance toward vasoconstriction and hypoperfusion. In the peripapillary region, altered microvascular autoregulation could similarly reduce measurable flow area without immediate structural loss.

RNFL thickness did not differ between groups. This negative finding is important, as it indicates preserved neural structure despite measurable perfusion alterations. Prior studies have reported heterogeneous RNFL findings in hypoestrogenic states (Deschênes et al., 2010; Fathy et al., 2022; Açmaz et al., 2014). Rather than indicating absence of estrogen-related neural effects, the preserved RNFL thickness in our cohort may reflect an early or subclinical stage of disease. This interpretation remains hypothetical and requires longitudinal confirmation.

CVI findings provide additional nuance. Macular CVI was reduced in POI, whereas peripapillary CVI did not differ significantly. This regional divergence may indicate that macular choroidal vascular-luminal proportion is more susceptible to early hypoestrogenic remodeling than the peripapillary choroid, potentially reflecting differences in vascular architecture and autoregulatory mechanisms. Further investigation is required to clarify this observation.

Approximately one-third of the superficial retina’s metabolic supply derives from the central retinal artery, whereas the majority of outer retinal oxygenation depends on the choroidal circulation (Archer, Gardiner & Stitt, 2007). The selective reduction in choroidal perfusion observed here, together with preserved RNFL thickness, supports the concept that vascular alterations may precede overt neurostructural damage in POI.

Because microvascular beds share common regulatory biology, OCTA-derived indices may reflect broader endothelial status. However, the proposal that choriocapillaris or ONH/RPC metrics function as systemic vascular surrogates remains speculative and was not directly tested in this study. Correlation with systemic endothelial markers and longitudinal outcomes will be necessary before such applications can be supported.

Methodological strengths include the first systematic OCTA assessment in POI, standardized imaging within a narrow time window, masked analyses, strict exclusion of systemic and ocular confounders, and multimodal evaluation of retinal, choroidal, and peripapillary vascular compartments.

Limitations include the cross-sectional, single-center design, which precludes causal inference and limits generalizability. The moderate sample size may increase the risk of type II error for subtle differences. Hormonal assays were not performed in controls; although regular menstrual cycles reduce the likelihood of occult estrogen deficiency, biochemical confirmation was not available. Additionally, systemic vascular biomarkers were not assessed, preventing direct correlation between ocular and systemic endothelial function.

## Conclusions

Women with premature ovarian insufficiency exhibit early ocular microvascular alterations characterized by reduced choriocapillaris flow, decreased subfoveal choroidal thickness, and diminished ONH/RPC flow areas, while retinal capillary density and RNFL thickness remain preserved.

This pattern suggests compartment-specific microvascular vulnerability in young hypoestrogenic women and supports the role of OCTA as a sensitive, hypothesis-generating tool for detecting subclinical vascular alterations in POI.

These findings should be interpreted within the constraints of a cross-sectional design and warrant validation in larger, longitudinal cohorts. Longitudinal and interventional studies incorporating detailed hormonal and systemic vascular assessment are warranted to determine temporal progression and clinical relevance.

## Supplemental Information

10.7717/peerj.21325/supp-1 Supplemental Information 1 Data

10.7717/peerj.21325/supp-2 Supplemental Information 2 STROBE checklist

## Additional Information and Declarations

### Data Availability

The following information was supplied regarding data availability: The raw measurements are available in the Supplementary File.

## References

[ref-1] Açmaz G, Ataş M, Gülhan A, Açmaz B, Ataş F, Aksoy H (2014). Evaluation of the macula, and layer, retinalnervefiber and choroid thickness in women with polycystic ovary syndrome using spectral-domain optical coherence tomography. Reproductive Sciences.

[ref-2] Agrawal R, Chhablani J, Tan K-A, Shah S, Sarvaiya C, Banker A (2016). Choroidal vascularity index in central serous chorioretinopathy. Retina.

[ref-3] Archer DB, Gardiner TA, Stitt AW (2007). Functional anatomy fine structure and basic pathology of the retinal vasculature. Retinal vascular disease.

[ref-4] Aryan L, Younessi D, Zargari M, Banerjee S, Agopian J, Rahman S, Borna R, Ruffenach G, Umar S, Eghbali M (2020). The role of estrogen receptors in cardiovascular disease. International Journal of Molecular Sciences.

[ref-5] Bedggood P, Metha A (2020). Adaptive optics imaging of the retinal microvasculature. Clinical and Experimental Optometry.

[ref-6] Çetinkaya Yaprak A, Erkan Pota Ç (2023). Comparison of retinochoroidal microvascular circulation in menstrual and postmenopausal periods using swept-source optical coherence tomography angiography. Graefe’s Archive for Clinical and Experimental Ophthalmology = Albrecht von Graefes Archiv fur klinische und experimentelle Ophthalmologie.

[ref-7] Chon SJ, Umair Z, Yoon M-S (2021). Premature ovarian insufficiency: past, present, and future. Frontiers in Cell and Developmental Biology.

[ref-8] Deschênes MC, Descovich D, Moreau M, Granger L, Kuchel GA, Mikkola TS, Fick GH, Chemtob S, Vaucher E, Lesk MR (2010). Postmenopausal hormone therapy increases retinal blood flow and protects the retinal nerve fiber layer. Investigative Ophthalmology & Visual Science.

[ref-9] Fathy M, Noureldine A, Elmofty HM, Tolba DA (2022). The effect of postmenopausal hormonal drop on optic nerve head and peripapillary perfusion using optical coherence tomography angiography (OCTA). Scientific Reports.

[ref-10] Goldsborough E, Osuji N, Blaha MJ, 51 (2022). Assessment of cardiovascular disease risk: a 2022 update. Endocrinology and Metabolism Clinics.

[ref-11] Guivarc’h E, Guihot A, Favre J, Vessières E, Wakim J, Arnal J, Lenfant F, Loufrani L, Henrion D (2015). CO-46: protective role of the estrogen receptor alpha during hypertension. Annales de Cardiologie et d’Angéiologie.

[ref-12] Ishizuka B (2021). Current understanding of the etiology, symptomatology, and treatment options in premature ovarian insufficiency (POI). Frontiers in Endocrinology.

[ref-13] Javed A, Khanna A, Palmer E, Wilde C, Zaman A, Orr G, Kumudhan D, Lakshmanan A, Panos GD (2023). Optical coherence tomography angiography: a review of the current literature. Journal of International Medical Research.

[ref-14] Joseph P, Leong D, McKee M, Anand SS, Schwalm J-D, Teo K, Mente A, Yusuf S (2017). Reducing the global burden of cardiovascular disease, part 1: the epidemiology and risk factors. Circulation Research.

[ref-15] Kellner RL, Harris A, Ciulla L, Guidoboni G, Verticchio Vercellin A, Oddone F, Carnevale C, Zaid M, Antman G, Kuvin JT, Siesky B (2024). The eye as the window to the heart: optical coherence tomography angiography biomarkers as indicators of cardiovascular disease. Journal of Clinical Medicine.

[ref-16] Matsunaga D, Yi J, Puliafito CA, Kashani AH (2014). OCT angiography in healthy human subjects. Ophthalmic Surgery, Lasers and Imaging Retina.

[ref-17] Matulevičiūė I, Sidaraitė A, Tatarūnas V, Veikutienė A, Dobilienė O, Žaliūnienė D (2022). Retinal and choroidal thinning—a predictor of coronary artery occlusion?. Diagnostics.

[ref-18] McGlacken-Byrne SM, Conway GS (2022). Premature ovarian insufficiency. Best Practice & Research Clinical Obstetrics & Gynaecology.

[ref-19] O’Leary F, Campbell M (2023). The blood–retina barrier in health and disease. The FEBS Journal.

[ref-20] Rezende GP, Dassie T, Gomes DAY, Benetti-Pinto CL (2023). Cardiovascular risk factors in premature ovarian insufficiency using hormonal therapy. Revista Brasileira de Ginecologia e Obstetrícia/RBGO Gynecology and Obstetrics.

[ref-21] Sumer F, Subasi S, Gurlek B, Ayazoglu I (2023). Meibography and tear function alterations in premature ovarian failure. Journal Français d’Ophtalmologie.

[ref-22] Vajaranant TS, Pasquale LR (2012). Estrogen deficiency accelerates aging of the optic nerve. Menopause.

[ref-23] Wickham LA, Gao J, Toda I, Rocha EM, Ono M, Sullivan DA (2000). Identification of androgen estrogen and progesterone receptor mRNAs in the eye. Acta Ophthalmologica Scandinavica.

[ref-24] Zhu Y, Bian Z, Lu P, Karas RH, Bao L, Cox D, Hodgin J, Shaul PW, Thorén P, Smithies O, Gustafsson JA, Mendelsohn ME (2002). Abnormal vascular function and hypertension in mice deficient in estrogen receptor *β*. Science.

