# Supplementary material for: Evaluation of retinal and choroidal microvasculature parameters by OCTA in patients with premature ovarian insufficiency: a prospective case control study
Source: PeerJ. 2026 Jun 29;14:e21325. doi: 10.7717/peerj.21325 (PMC13326647; doi:10.7717/peerj.21325)
Supplement: Supplemental Information 2 [file peerj-14-21325-s002.docx]

STROBE Statement – Checklist for Case-Control Studies

## Item 1: 1. Title and abstract

The title and abstract indicate the study design by mentioning 'prospective case-control study' and summarize key findings related to ocular microvascular alterations in women with POI.

## Item 2: 2. Background/rationale

The introduction provides a clear explanation of the scientific background and rationale, discussing the role of estrogen in vascular health and ocular structures, and the lack of OCT-A based studies in POI.

## Item 3: 3. Objectives

The study objective is explicitly stated: to investigate ocular microvascular characteristics using OCT-A in women diagnosed with POI and compare them to healthy controls.

## Item 4: 4. Study design

Key elements of the study design (prospective case-control) are presented early in the methods section.

## Item 5: 5. Setting

The study was conducted at the Ophthalmology and Gynecology Departments of Kahramanmaraş Sütçü İmam University Hospital from January 2021 to January 2023.

## Item 6: 6a. Participants

37 women with POI and 50 healthy controls were included. POI diagnosis was based on amenorrhea/oligomenorrhea and elevated FSH levels. Exclusion criteria and rationale for control selection are described.

## Item 7: 6b. Matched studies

Not applicable. Although controls were age-matched, no formal matching criteria were used.

## Item 8: 7. Variables

Outcomes, exposures, and confounders (e.g., hormonal status, age, BMI) are clearly defined. Diagnostic criteria for POI and details of ocular parameters are provided.

## Item 9: 8. Data sources/measurement

Sources and measurement methods are detailed for each variable. OCT-A parameters and ocular assessments are explained, with emphasis on consistency across study groups.

## Item 10: 9. Bias

Efforts to reduce bias include standardized imaging protocols, morning measurement sessions, and blinded assessment of images by a single investigator.

## Item 11: 10. Study size

No formal sample size calculation was performed a priori; the sample was determined by participant availability during the study period.

## Item 12: 11. Quantitative variables

Continuous variables were analyzed as means ± SD. No categorization of quantitative variables is reported.

## Item 13: 12a. Statistical methods

Student’s t-test and chi-square tests were used to compare groups. P-values < 0.05 were considered significant.

## Item 14: 12b. Subgroups and interactions

No subgroup or interaction analyses were conducted.

## Item 15: 12c. Missing data

No missing data were reported.

## Item 16: 12d. Matching

Not applicable.

## Item 17: 12e. Sensitivity analyses

Not performed.

## Item 18: 13a. Participants

A total of 60 POI candidates and 77 healthy controls were initially considered. After exclusions, 37 POI and 50 controls were analyzed.

## Item 19: 13b. Reasons for non-participation

Exclusions were due to criteria such as chronic systemic or ocular disease or medication use.

## Item 20: 13c. Flow diagram

A flow diagram was created to show the participant selection process.

## Item 21: 14a. Descriptive data

Demographic and clinical characteristics, including age, BMI, gravida, parity, and ocular surface tests, are reported in Table 1.

## Item 22: 14b. Missing data

No missing data were identified.

## Item 23: 15. Outcome data

Key outcome measures (OCT-A parameters, choroidal thickness, flow density) are summarized in Tables 2 and 3.

## Item 24: 16a. Main results

Unadjusted p-values are reported. No confounder-adjusted estimates were calculated.

## Item 25: 16b. Category boundaries

Not applicable as continuous variables were used.

## Item 26: 16c. Translating estimates

Not applicable.

## Item 27: 17. Other analyses

No subgroup or sensitivity analyses were conducted.

## Item 28: 18. Key results

The discussion highlights significantly reduced choroidal thickness, choriocapillaris flow, and optic nerve head perfusion in POI patients.

## Item 29: 19. Limitations

Limitations include small sample size, cross-sectional design, and lack of hormonal data correlation.

## Item 30: 20. Interpretation

The discussion provides a cautious interpretation, relating findings to the hypothesis of hypoestrogenic microvascular impact, supported by previous studies.

## Item 31: 21. Generalisability

Generalizability may be limited due to the single-center design and demographic homogeneity.

## Item 32: 22. Funding

This study received no specific funding from public, commercial, or non-profit sectors.
